# Single nucleotide polymorphisms in the FAM167A-BLK gene are associated with polymyositis/dermatomyositis in the Han Chinese population

**DOI:** 10.1007/s12026-015-8646-0

**Published:** 2015-04-07

**Authors:** Si Chen, Wei Wu, Jing Li, Qian Wang, Yuan Li, Ziyan Wu, Wenjie Zheng, Qingjun Wu, Chanyuan Wu, Fengchun Zhang, Yongzhe Li

**Affiliations:** Department of Rheumatology and Clinical Immunology, Peking Union Medical College Hospital, Chinese Academy of Medical Sciences & Peking Union Medical College, Key Laboratory of Rheumatology and Clinical Immunology, Ministry of Education, 41 Damucang Hutong, Xicheng District, Beijing, 100032 China; Department of Clinical Laboratory, Peking Union Medical College Hospital, Peking Union Medical College and Chinese Academy of Medical Sciences, Beijing, China

**Keywords:** Dermatomyositis, Polymyositis, FAM167A-BLK, Polymorphism, Association

## Abstract

Idiopathic inflammatory myopathies (IIMs) are autoimmune diseases influenced by genetic background and environmental factors. Recently, FAM167A-BLK gene has been identified as a potential genetic susceptibility locus for dermatomyositis (DM) in patients of European and Japanese populations. Our aim here was to investigate the association between FAM167A-BLK polymorphisms and IIMs risk in Chinese Han. The FAM167A-BLK single nucleotide polymorphisms (SNPs) rs2736340, rs7812879, rs13277113, rs2618479, rs2254546 and rs2248932 were analyzed in polymyositis (PM) patients (*n* = 310), DM patients (*n* = 535) and 968 ethnically matched healthy controls, with the Sequenom MassArray system. Our present study demonstrated that strong allele association was observed in overall PM/DM and PM patients for rs2736340 (*P*_c_ = 6.48 × 10^−3^; *P*_c_ = 0.013, respectively), rs7812879 (*P*_c_ = 0.017; *P*_c_ = 0.034, respectively) and rs13277113 (*P*_c_ = 0.011; *P*_c_ = 0.047, respectively). These three SNPs were significantly associated with interstitial lung disease (ILD) in overall PM/DM patients (all, *P*_c_ < 0.05). The frequency of the five haplotypes of the five SNPs (rs2736340, rs7812879, rs13277113, rs2618479 and rs2254546) was also significantly different between overall PM/DM, PM or DM patients and healthy controls. This was the first study to demonstrate that the FAM167A-BLK polymorphisms were associated with Chinese PM/DM patients or these patients with ILD, indicating that PM/DM might share common gene with other autoimmune diseases.

## Introduction

The idiopathic inflammatory myopathies (IIMs), collectively named myositis, are characterized by symmetrical, proximal muscle weakness and inflammation in skeletal muscle tissue. Based on their clinical and histopathological features, they may be subdivided into polymyositis (PM), dermatomyositis (DM), inclusion body myositis (IBM) and myositis overlapping with other connective tissue diseases (myositis–CTD overlap syndrome). Besides, PM and DM are the most common subtypes. IIMs are a rare disease in the Caucasian population, with an overall annual prevalence of approximately 10–15 cases per 100,000 individuals [1]. However, its incidence in the Chinese population remains unclear due to the lack of large-scale epidemiological studies. Although IIMs are generally considered as complex multifactorial rheumatic disease, the etiology of IIMs is mostly unknown.

It is supposed that the development of IIMs may be the result of both genetic and environmental factors or their interactions. Since IIMs are a rare autoimmune disease, the development of their genetic studies had been restrained [1]. To date, the strongest genetic associations in the IIMs have consistently been shown to be within the major histocompatibility complex (MHC) gene region [2], whereas only a touch of non-MHC loci was identified by genome-wide association study (GWAS) and candidate gene association studies. Non-MHC genes such as tumor necrosis factor alpha (TNF-α) [3–6], interleukin (IL)-1α, IL-1β [5], interferon (IFN)-γ [7], interferon-induced helicase (IFIH1) [8], mannose-binding lectin 2 (MBL2) [9], protein tyrosine phosphatase N22 (PTPN22) [10], and signal transducer and activator of transcription 4 (STAT4) [11] confer susceptibility to PM/DM, which indicated that PM/DM shared genetic susceptibility with other autoimmune diseases.

The GWAS [12] had been undertaken on European DM patients. This was the first GWAS with regard to DM, and it revealed that DM share enrichment of genetic loci with other autoimmune diseases. This study confirmed involvement of the MHC region on chromosome 6, although no loci outside the MHC reached genome-wide significance [*P* ≤ 5 × 10^−8^]. These results identified three genes possibly associated with DM: phospholipase C-like 1 (PLCL1), B lymphoid tyrosine kinase (BLK) and chemokine (C–C motif) ligand 21 (CCL21), none of which had been reported previously in DM. The family with sequence similarity 167 member A-B-lymphoid tyrosine kinase (FAM167A-BLK) region has been well confirmed as susceptibility factors for several autoimmune diseases. Recently, Jani et al. [13] indicated that two SNPs of FAM167A-BLK (rs13277113 and rs2618476) were associated with DM but not PM. Besides, Sugiura et al. [14] showed that rs13277113 in FAM167A-BLK gene was associated with Japanese IIMs patients. In addition, six variants rs2736340, rs7812879, rs13277113, rs2618479, rs2254546 and rs2248932 in the intergenic region between the FAM167A and BLK genes are the common risk factors for systemic lupus erythematosus (SLE) [15–17], rheumatoid arthritis (RA) [18, 19], systemic sclerosis (SSc) [20] and primary sjögren’s syndrome (pSS) [21, 22] identified by GWAS and candidate gene association studies in Caucasian and Asian populations. Moreover, upregulation of B cell has been observed in IIMs patients [23], and increased evidence has suggested that auto-reactive B cells play an important role in the pathogenesis of IIMs [24]. Based on FAM167A-BLK gene is involved in the activation and function of B cell, we designed a case–control study to explore the hypothesis that variants of FAM167A-BLK gene might predispose to PM/DM in a Chinese Han population. Our aim was to disclose population differences in genetic variations related in the pathogenesis of PM/DM to improve clinical interference of this disease.

## Methods

### Subjects

Our research was designed as a large cross-sectional study, and a total of 1819 subjects consisting of 851 PM/DM patients and 968 healthy controls ethnically matched to the cases were recruited in current study. These patients were enrolled from two different sources. Between February 2013 and July 2014, 473 patients including 155 PM patients and 318 DM patients were enrolled from the Peking Union Medical College Hospital. Since this study was supported by the Research Special Fund for Public Welfare Industry of Health, 378 patients containing 155 PM patients and 223 DM patients were recruited through the cooperation of three centers in China.

All patients were ≥18 years at the onset of disease and had probable/definite myositis valuated by at least two rheumatologists in light of the criteria of Bohan and Peter [25, 26]. Patients with myositis–CTD overlap syndrome were excluded if they met any of the following published criteria: American College of Rheumatology (ACR) criteria for SLE [27], RA [28], and SSc [29], the American and European consensus criteria for pSS [30] or the criteria for mixed CTD by Sharp et al. [31]. At the same time, we excluded amyopathic dermatomyositis (ADM), who could not meet the traditional criteria of Sontheimer [32]. IBM patients and patients with muscle diseases caused by other factors were systematically excluded. A total of 968 ethnically matched healthy controls were collected from the Peking Union Medical College Hospital during their physical examinations. Our study was approved by the Ethics Committee of the Peking Union Medical College Hospital, and all participants signed a written informed consent.

### Selection of SNPs

Given the significant functions of FAM167A-BLK in multiple autoimmune diseases, six SNPs (rs2736340, rs7812879, rs13277113, rs2618479, rs2254546 and rs2248932) of FAM167A-BLK gene, which had previously demonstrated in positive associations with other immune-mediated diseases based on GWAS or candidate gene association studies, were examined in our present study.

### Genotyping

Peripheral blood specimen (2 mL) was collected from patients and controls, and DNA of each participant was extracted from peripheral white blood cell by using kits from Tiangen (Beijing, China) and complied with the manufacturer’s instructions. The genotyping of all six SNPs from FAM167A-BLK gene was performed using a Sequenom MassArray system (Sequenom iPLEX assay, San Diego, CA, USA) in accordance with the manufacturer’s protocol. The primers for polymerase chain reaction (PCR) and for locus-specific single-base extension were designed by the MassArray Assay Design 4.0 (Sequenom). All DNA samples of patients and controls were first transferred to a 384-element plate. After multiplex PCR amplifications, the products were used for locus-specific single-base extension reactions. The final products were subsequently desalted and transferred to 384-element SpectroCHIP array (Sequenom). Allele detection was carried out by matrix-assisted laser desorption/ionization-time-of-flight mass spectrometry (MALDI-TOF MS). The resultant mass spectrograms and genotype data were analyzed using MassArray Typer 4.0 software.

### Statistical analysis

The Hardy–Weinberg equilibrium (HWE) of each SNP was examined by using the Chi-square (*χ*^2^) test in healthy controls, and any SNPs that deviated from the HWE (*P* < 0.05 in the control groups) would be excluded from subsequent analysis. Differences in genotype and allele frequencies between cases and controls were analyzed by the *χ*^2^ test. The odds ratio (OR) and 95 % confidence interval (95 % CI) of associations were calculated, and *P* values (corrected for multiple comparisons by the Bonferroni adjustment test) <0.05 were deemed to be statistically significant. For genetic model testing (additive, dominant and recessive model), genotype frequencies were further analyzed using logistic regression models. For the association analysis between FAM167A-BLK polymorphisms and the three clinical subsets (overall PM/DM patients, PM patients and DM patients versus control groups), statistical analysis was calculated by PLINK v1.07 software (Shaun Purcell, Boston, USA) [33]. Stratification analysis with regard to the association study for these six SNPs and the presence of interstitial lung disease (ILD) were performed by the results of the following three comparisons: patients (overall PM/DM patients, PM patients and DM patients) with ILD versus all controls, patients without ILD versus all controls and patients with ILD versus without ILD. The genetic power for this large cross-sectional study was accomplished with the statistical program Genetic Power Calculator [34]. Haplotype analysis was performed with Haploview software v4.2 [35].

## Results

### The characteristics of subjects

In the present study, a total of 851 adult-onset PM/DM patients (74.5 % women; mean age 45.6 ± 14.9) were enrolled, including 310 PM patients (73.4 % women; mean age 45.2 ± 14.7) and 535 DM patients (75.6 % women; mean age 46.0 ± 15.1). The ethnically matched healthy controls included 968 subjects (83.7 % women; mean age 43.4 ± 12.8 years). For these patients, 171 of 310 PM patients (55.2 %) and 308 of 541 DM patients (56.9 %) had complicated with ILD. Finally, 479 PM/DM patients had complicated with ILD and 372 patients not. Characteristics of cases and controls engaged in present study are summarized in Table 1. All SNPs in FAM167A-BLK gene were in Hardy–Weinberg equilibrium in controls (*P*_HWE_ > 0.05). The genotype call rates of each SNP were >97 %. The power analysis showed that our sample size had more than 80 % power (*α* = 0.05) for detecting association with an OR of 1.10–1.60 for both heterozygotes and homozygotes.

**Table 1:** Clinical data for PM/DM patients and controls

Characteristic	Patients	Controls
Number of subjects (DM/PM)	851 (535/310)	968
Female ratio (%)	74.5	83.7
Average age	45.6 ± 14.9	43.4 ± 12.8
DM with ILD, No./total (%)	308/541 (56.9)	–
PM with ILD, No./total (%)	171/310 (55.2)	–

*PM* polymyositis, *DM* dermatomyositis, *ILD* interstitial lung disease

### Association of the six SNPs with PM/DM in the Han population

Table 2 summarizes the genotype and allele distribution for the six SNPs in FAM167A-BLK gene. There was statistically significant difference in the allelic and genotypic frequencies in the rs2736340 between overall PM/DM patients and healthy controls (*P*_c_ = 6.48 × 10^−3^ and *P*_c_ = 0.032; Table 2). In addition, we discovered that the rs2736340 allele was associated with PM development (*P*_c_ = 0.013). Also, the allele frequencies of rs7812879 and rs13277113 showed statistically significant associations with PM patients and overall PM/DM patients (*P*_c_ = 0.034 and *P*_c_ = 0.017; *P*_c_ = 0.047 and *P*_c_ = 0.011, respectively). The percentage of overall PM/DM patients with C allele of rs2618479 was significantly higher than that in the healthy controls (*P*_c_ = 0.040). However, neither of other two SNPs (rs2254546 and rs2248932) demonstrated significant differences in allele or genotype frequencies between patients and controls (all, *P*_c_ > 0.05).

**Table 2:** Allele and genotype distribution of the BLK gene markers in PM/DM patients and controls

SNPs	Groups	Allele (%)		OR (95 % CI)	*P*	*P*_c_	Genotype (%)			*χ* ^2^	*P*	*P*_c_
		C	T				CC	CT	TT			
rs2736340	PM	123 (20.2)	487 (79.8)	0.71 (0.57–0.88)	2.24 × 10^−3^	**0.013**	11 (3.6)	101 (33.1)	193 (63.3)	9.41	9.03 × 10^−3^	0.054
	DM	239 (22.5)	825 (77.5)	0.81 (0.68–0.97)	0.020	0.120	30 (5.6)	179 (33.7)	323 (60.7)	5.40	0.067	0.402
	PM + DM	362 (21.6)	1312 (78.4)	0.77 (0.66–0.90)	1.08 × 10^−3^	**6.48** **×** **10** ^**−3**^	31 (3.7)	280 (33.9)	516 (62.4)	10.5	5.28 × 10^−3^	**0.032**
	Controls	508 (26.3)	1424 (73.7)				70 (7.2)	368 (38.1)	528 (54.7)			
		T	C				TT	TC	CC			
rs7812879	PM	105 (17.3)	503 (82.7)	0.72 (0.57–0.91)	6.74 × 10^−3^	**0.034**	8 (2.6)	89 (29.3)	207 (68.1)	7.54	0.023	0.138
	DM	202 (19.0)	860 (81.0)	0.81 (0.67–0.98)	0.029	0.174	25 (4.7)	152 (28.6)	354 (66.7)	4.99	0.082	0.492
	PM + DM	307 (18.4)	1363 (81.6)	0.78 (0.66–0.92)	2.79 × 10^−3^	**0.017**	33 (4.0)	241 (28.8)	561 (67.2)	8.61	0.014	0.084
	Controls	434 (22.4)	1502 (77.6)				55 (5.7)	324 (33.5)	589 (60.8)			
		G	A				GG	GA	AA			
rs13277113	PM	137 (22.5)	473 (77.5)	0.75 (0.60–0.93)	7.77 × 10^−3^	**0.047**	14 (4.6)	109 (35.7)	182 (59.7)	7.23	0.027	0.162
	DM	252 (23.9)	804 (76.1)	0.81 (0.68–0.96)	0.016	0.096	35 (6.6)	182 (34.5)	311 (58.9)	6.06	0.048	0.288
	PM + DM	389 (23.3)	1277 (76.7)	0.79 (0.68–0.91)	1.77 × 10^−3^	**0.011**	49 (5.9)	291 (34.9)	493 (59.2)	9.58	8.30 × 10^−3^	0.050
	Controls	540 (27.9)	1394 (72.1)				79 (8.2)	382 (39.5)	506 (52.3)			
		T	C				TT	TC	CC			
rs2618479	PM	124 (20.4)	484 (79.6)	0.77 (0.62–0.97)	0.023	0.138	10 (3.3)	104 (34.2)	190 (62.5)	5.76	0.056	0.336
	DM	228 (21.5)	834 (78.5)	0.82 (0.69–0.99)	0.035	0.210	31 (5.8)	166 (31.3)	334 (62.9)	5.94	0.051	0.306
	PM + DM	352 (21.1)	1318 (78.9)	0.81 (0.69–0.94)	6.69 × 10^−3^	**0.040**	41 (4.9)	270 (32.3)	524 (62.8)	7.50	0.023	0.138
	Controls	482 (24.9)	1454 (75.1)				61 (6.3)	360 (37.2)	547 (56.5)			
		A	G				AA	AG	GG			
rs2254546	PM	111 (18.2)	499 (81.8)	0.77 (0.61–0.97)	0.027	0.162	11 (3.6)	89 (29.2)	205 (67.2)	4.75	0.093	0.558
	DM	215 (20.2)	851 (79.8)	0.87 (0.73–1.05)	0.152	0.912	31 (5.8)	153 (28.7)	349 (65.5)	3.39	0.184	1.104
	PM + DM	326 (19.5)	1350 (80.5)	0.84 (0.71–0.98)	0.029	0.174	42 (5.0)	242 (28.9)	554 (66.1)	5.15	0.076	0.456
	Controls	434 (22.4)	1502 (77.6)				56 (5.8)	322 (33.3)	590 (60.9)			
		C	T				CC	CT	TT			
rs2248932	PM	130 (21.3)	480 (78.7)	0.86 (0.69–1.07)	0.165	0.990	8 (2.6)	114 (37.4)	183 (60.0)	4.88	0.087	0.522
	DM	239 (22.5)	823 (77.5)	0.92 (0.77–1.10)	0.342	2.052	31 (5.8)	177 (33.3)	323 (60.9)	1.55	0.462	2.772
	PM + DM	369 (22.1)	1303 (77.9)	0.89 (0.77–1.05)	0.160	0.960	39 (4.7)	291 (34.8)	506 (60.5)	2.05	0.360	2.160
	Controls	465 (24.0)	1469 (76.0)				56 (5.8)	353 (36.5)	558 (57.7)			

Bold values are statistically significant (*P* < 0.05) (corrected for multiple comparisons by the Bonferroni adjustment test)

*PM* polymyositis, *DM* dermatomyositis, *OR* odds ratio, *CI* confidence interval, *χ* ^2^ Chi-square test, *P*_c_ *P* value corrected by Bonferroni method

Further logistic regression analysis was accomplished based upon three genetic models (additive, dominant and recessive model). The outcomes of logistic regression analysis are summarized in Table 3. In the additive and the dominant model, significant associations were observed in PM patients and overall PM/DM patients for rs2736340 (*P*_c_ = 0.015 and *P*_c_ = 7.43 × 10^−3^; *P*_c_ = 0.049 and *P*_c_ = 0.016, respectively). Besides, associations were observed under the additive and the dominant model for three SNPs (rs7812879, rs13277113 and rs2618479) in overall PM/DM patients (all, *P*_c_ < 0.05). In addition, rs7812879 indicated weak association with PM patients in the additive model (*P*_c_ = 0.047). Nevertheless, none of the three genetic models showed any significant differences between patients and controls for two SNPs (rs2254546 and rs2248932) (all, *P*_c_ > 0.05).

**Table 3:** Analysis of the six SNPs based on three genetic models

SNPs	Groups	Additive model		Dominant model		Recessive model	
		*P*_c_	OR (95 % CI)	*P*_c_	OR (95 % CI)	*P*_c_	OR (95 % CI)
rs2736340	PM	**0.015**	0.71 (0.57–0.89)	**0.049**	0.70 (0.54–0.91)	0.157	0.47 (0.25–0.91)
	DM	0.133	0.82 (0.69–0.97)	0.142	0.78 (0.63–0.97)	1.405	0.76 (0.49–1.19)
	PM + DM	**7.43** **×** **10** ^**−3**^	0.78 (0.67–0.91)	**0.016**	0.75 (0.62–0.91)	0.239	0.66 (0.44–0.98)
rs7812879	PM	**0.047**	0.73 (0.58–0.92)	0.138	0.73 (0.55–0.96)	0.222	0.45 (0.21–0.95)
	DM	0.204	0.82 (0.68–0.99)	0.155	0.78 (0.62–0.97)	2.539	0.82 (0.50–1.33)
	PM + DM	**0.021**	0.79 (0.67–0.92)	**0.032**	0.76 (0.63–0.92)	0.545	0.68 (0.44–1.06)
rs13277113	PM	0.05	0.75 (0.61–0.93)	0.150	0.74 (0.57–0.96)	0.234	0.54 (0.30–0.97)
	DM	0.109	0.81 (0.69–0.97)	0.089	0.77 (0.62–0.95)	1.705	0.80 (0.53–1.21)
	PM + DM	**0.012**	0.79 (0.68–0.92)	**0.021**	0.76 (0.63–0.91)	0.365	0.70 (0.49–1.02)
rs2618479	PM	0.138	0.77 (0.62–0.96)	0.391	0.78 (0.60–1.02)	0.300	0.51 (0.26–1.00)
	DM	0.226	0.83 (0.69–0.99)	0.098	0.77 (0.62–0.95)	4.324	0.92 (0.59–1.44)
	PM + DM	**0.044**	0.81 (0.69–0.94)	**0.043**	0.77 (0.64–0.93)	1.220	0.77 (0.51–1.15)
rs2254546	PM	0.182	0.78 (0.62–0.98)	0.300	0.76 (0.58–1.00)	0.846	0.61 (0.32–1.18)
	DM	0.995	0.88 (0.74–1.05)	0.498	0.82 (0.66–1.03)	5.882	1.01 (0.64–1.58)
	PM + DM	0.206	0.84 (0.72–0.99)	0.140	0.80 (0.66–0.97)	2.819	0.86 (0.57–1.30)
rs2248932	PM	0.959	0.85 (0.68–1.07)	2.871	0.91 (0.70–1.18)	0.189	0.44 (0.21–0.93)
	DM	2.076	0.92 (0.77–1.10)	1.440	0.88 (0.71–1.09)	5.822	1.01 (0.64–1.59)
	PM + DM	0.961	0.89 (0.76–1.05)	1.346	0.89 (0.74–1.07)	1.721	0.80 (0.52–1.21)

Bold values are statistically significant (*P* < 0.05) (corrected for multiple comparisons by the Bonferroni adjustment test)

*PM* polymyositis, *DM* dermatomyositis, *OR* odds ratio, *CI* confidence interval, *P*_c_ *P* value corrected by Bonferroni method

### Association between FAM167A-BLK polymorphisms and the ILD phenotype of PM/DM

To analyze FAM167A-BLK polymorphisms in more detail, we further observed whether the associations existed between FAM167A-BLK polymorphisms and ILD phenotype of PM/DM patients. The associations between the six SNPs and patients with/without ILD are shown in Table 4. Notably, the four SNPs (rs2736340, rs7812879, rs13277113 and rs2618479) were associated with overall PM/DM patients with ILD involvement (all, *P*_c_ < 0.05). However, our present study indicated that the other two SNPs (rs2254546 and rs2248932) were not statistically significantly associated with PM/DM patients with/without ILD in present study (Table 4).

**Table 4:** Association between the six SNPs and PM/DM with ILD

Disease	Groups	rs2736340		rs7812879		rs13277113		rs2618479		rs2254546		rs2248932	
		*P*_c_	OR (95 % CI)	*P*_c_	OR (95 % CI)	*P*_c_	OR (95 % CI)	*P*_c_	OR (95 % CI)	*P*_c_	OR (95 % CI)	*P*_c_	OR (95 % CI)
PM	P versus N	4.89	0.95 (0.64–1.41)	3.08	0.87 (0.57–1.33)	3.87	0.91 (0.62–1.34)	2.85	0.87 (0.58–1.29)	5.13	0.96 (0.64–1.46)	3.80	1.10 (0.74–1.63)
	P versus C	0.07	0.69 (0.52–0.92)	0.08	0.68 (0.50–0.92)	0.11	0.72 (0.54–0.95)	0.17	0.72 (0.54–0.97)	0.39	0.76 (0.56–1.02)	2.53	0.89 (0.68–1.18)
	N versus C	0.26	0.73 (0.53–0.99)	0.79	0.78 (0.56–1.08)	0.68	0.79 (0.58–1.06)	1.50	0.84 (0.62–1.14)	0.88	0.79 (0.57–1.09)	1.13	0.81 (0.59–1.11)
DM	P versus N	2.40	0.88 (0.66–1.18)	2.31	0.87 (0.64–1.19)	1.41	0.84 (0.63–1.12)	2.65	0.89 (0.66–1.20)	4.81	0.96 (0.71–1.30)	3.30	0.92 (0.68–1.22)
	P versus C	0.11	0.77 (0.62–0.96)	0.15	0.77 (0.61–0.97)	0.05	0.75 (0.60–0.93)	0.19	0.78 (0.63–0.98)	1.14	0.86 (0.69–1.08)	1.59	0.88 (0.71–1.10)
	N versus C	1.51	0.87 (0.69–1.10)	1.84	0.88 (0.68–1.13)	2.00	0.89 (0.71–1.13)	1.81	0.88 (0.69–1.12)	2.28	0.89 (0.70–1.15)	4.58	0.96 (0.76–1.22)
PM + DM	P versus N	2.53	0.91 (0.72–1.15)	1.70	0.87 (0.68–1.12)	1.34	0.87 (0.69–1.09)	1.80	0.88 (0.70–1.12)	4.59	0.96 (0.76–1.23)	5.11	0.98 (0.78–1.23)
	P versus C	**0.009**	0.74 (0.61–0.89)	**0.01**	0.73 (0.60–0.90)	**0.007**	0.74 (0.62–0.89)	**0.03**	0.76 (0.63–0.92)	0.29	0.82 (0.68–1.00)	1.22	0.88 (0.74–1.07)
	N versus C	0.28	0.82 (0.67–1.00)	0.64	0.84 (0.68–1.04)	0.64	0.85 (0.70–1.04)	0.93	0.86 (0.71–1.06)	0.85	0.85 (0.69–1.05)	2.03	0.91 (0.74–1.11)

Bold values are statistically significant (*P* < 0.05) (corrected for multiple comparisons by the Bonferroni adjustment test)

*DM* dermatomyositis, *PM* polymyositis, *ILD* interstitial lung disease, *OR* odds ratio, *CI* confidence interval, *P*_c_ *P* value corrected by Bonferroni method

Group P: patients with ILD; Group N: patients without ILD; Group C: healthy controls; Group P (PM: *n* = 171; DM: *n* = 308; PM + DM: *n* = 479); Group N (PM: *n* = 139; DM: *n* = 233; PM + DM: *n* = 372); Group C (*n* = 968)

### Haplotype analysis of TNFAIP3 SNPs and patients

**Fig. 1:**
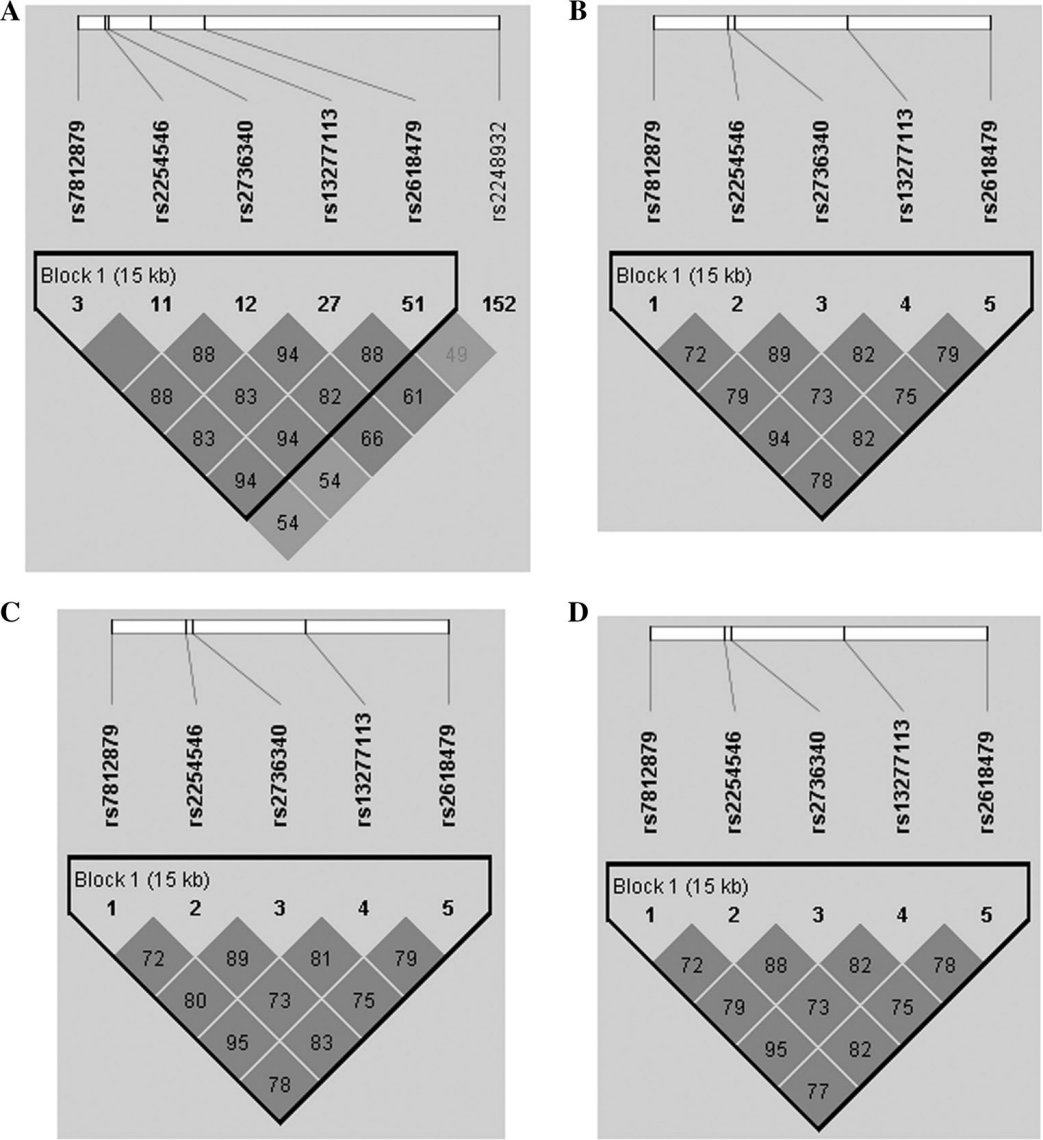
Linkage disequilibrium (LD) analysis of the SNPs in the FAM167A-BLK gene region. The LD plots were generated by Haploview software v4.2, and data from our study were similar to that from the HapMap CHB population. The number (divided by 100) in the *small square* represents *r* ^2^ value and ranges from 0 to 1. The five SNPs (rs2736340, rs7812879, rs13277113, rs2618479 and rs2254546) in FAM167A-BLK reside in an LD block. **a** The data from HapMap CHB. **b** The data analysis between PM patients and healthy controls from our study. **c** The data analysis between DM patients and healthy controls from our study. **d** The data analysis between PM/DM patients and healthy controls from our study

We used Haploview software to further analyze the distributions of the haplotypes in the FAM167A-BLK SNPs between patients and healthy controls. The results from the LD analysis of the SNPs (rs2736340, rs7812879, rs13277113, rs2618479 and rs2254546) in our study and the data from the HapMap CHB population are shown in Table 5 and Fig. 1. Data from HapMap CHB and the present study illustrated no significant differences. The TGCAT haplotype (rs2736340T–rs7812879G–rs13277113C–rs2618479A–rs2254546T) had a lower frequency between PM, DM or PM/DM patients and healthy controls (*P*_c_ = 0.0068, *P*_c_ = 0.0181 and *P*_c_ = 2.0 × 10^−3^, respectively; Table 5).

**Table 5:** Haplotype analysis of BLK SNPs between DM patients and controls

Groups	Haplotypes					Total of frequency (%)	Case (%)	Control (%)	*χ* ^2^	*P*_c_
	rs7812879	rs2254546	rs2736340	rs13277113	rs2618479					
PM	C	A	T	G	C	73.8	74.0	72.1	6.29	**0.0122**
	T	G	C	A	T	20.9	17.5	22.1	7.33	**0.0068**
	C	G	C	G	C	2.7	0.8	2.9	6.67	**0.0098**
	C	G	T	G	T	1.5	1.9	1.8	0.13	0.7203
	C	G	C	G	T	1.1	1.8	1.1	0.20	0.6574
DM	C	A	T	G	C	73.8	76.2	72.2	4.15	**0.0415**
	T	G	C	A	T	20.8	18.3	21.9	5.58	**0.0181**
	C	G	C	G	C	2.5	1.9	2.7	2.73	0.0986
	C	G	T	G	T	1.8	1.8	1.9	0.01	0.922
	C	G	C	G	T	1.2	1.8	1.3	0.51	0.4729
PM + DM	C	A	T	G	C	74.3	76.4	72.1	8.18	**0.042**
	T	G	C	A	T	20.1	17.9	21.9	9.53	**0.002**
	C	G	C	G	C	2.3	1.8	2.8	7.15	**0.0075**
	C	G	T	G	T	1.8	2.0	1.9	0.06	0.8079
	C	G	C	G	T	1.5	1.9	1.3	0.54	0.4625

Bold values are statistically significant (*P* < 0.05) (corrected for multiple comparisons by the Bonferroni adjustment test)

*PM* polymyositis, *DM* dermatomyositis, *P*_c_ *P* value corrected by Bonferroni method

## Discussion

Herein, we first described the association between FAM167A-BLK polymorphisms and PM/DM patients in a Chinese Han population. Our study confirmed that the four SNPs (rs2736340, rs7812879, rs13277113 and rs2618479) in FAM167A-BLK gene region were associated with PM/DM in Chinese Han. It is interesting that even after stratification into traditional IIMs clinical subgroups, the association with PM also presented, but not in patients with DM. The association between FAM167A-BLK polymorphisms (rs2736340, rs13277113 and rs2618479) and IIMs patients was also conducted in Caucasian population [12, 13]. However, Jani et al. [13] indicated that two SNPs of FAM167A-BLK (rs13277113 and rs2618476) were associated with DM but not PM, and the GWAS undertaken on European DM patients also demonstrated that rs2736340 was associated with Caucasian DM patients [12]. Moreover, Sugiura et al. [14] showed that rs13277113 in FAM167A-BLK gene was associated with Japanese IIMs patients, especially in DM patients. Based on the above research and our study, we found the role of FAM167A-BLK gene played in the pathogenesis of DM and PM existed racial and regional differences, which assisted us to improve the clinical interference of this disease.

PM is characterized by symmetric proximal muscle weakness, whereas DM is characterized by an erythematous rash and symmetric proximal muscle weakness. PM and DM are the major clinical subtypes of IIMs, and studies had manifested that the immunopathology of PM and DM is different. DM patients’ muscle biopsy demonstrates a mononuclear, inflammatory cell exudate arranged in predominantly perivascular or in the interfascicular septae rather than within the fascicles [36, 37]. On the contrary, PM has a distinct immunohistopathologic phenotype, in which muscle biopsy reveals that the primary inflammatory cell is lymphocytes (CD8-positive cells) invaded histologically healthy muscle fibers expressing MHC class I antigens [36–38]. Our present study suggested that the role of FAM167A-BLK gene played in the pathogenesis of DM and PM is distinct, presumably because the above immunopathogenesis of PM and DM is inconsistent. At the same time, our study showed that FAM167A-BLK gene polymorphisms were associated with combined cases. In fact, this may be because FAM167A-BLK gene polymorphisms might have high sensitivity for Chinese PM patients, and the relationship between FAM167A-BLK gene polymorphisms and PM patients was found on condition that the sample size of PM patients in our present study was small. Whereas FAM167A-BLK gene polymorphisms might have low sensitivity for Chinese DM patients, the relationship between FAM167A-BLK gene polymorphisms and DM patients was not found on condition that the sample size of PM patients in our present study was relatively large. Interestingly, when we combined the patients, the association with PM/DM was strong, which owing to the larger sample size. Future studies about PM/DM patients utilizing larger numbers of patients should be executed to confirm these consequences.

The FAM167A is also known as C8orf13, yet the function of FAM167A remains a mystery. However, the BLK is known to encode a non-receptor tyrosine kinase of the src family that is mainly expressed by B cells [39]. The BLK has a role in B cell receptor (BCR) signaling and B cell development by activating the nuclear factor (NF)-kappa B [40]. BCR signaling requires a tight regulation of several protein tyrosine kinases and phosphatases, and associated co-receptors. Binding of antigen to the BCR triggers signaling, which ultimately leads to B cell activation [40]. In the current study, we found that the rs2736340, rs7812879, rs13277113 and rs2618479 variants were associated with the development of PM/DM in Chinese Han population and extended previous finding in other rheumatic diseases, such as SLE [15–17], RA [18, 19], SSc [20] and pSS [21, 22]. Therefore, our study confirmed that PM/DM shares a gene commonly associated with the risk of other autoimmune diseases. Thus, FAM167A-BLK gene may in fact play a foremost role in the pathogenesis of multiple autoimmune diseases as well as to PM/DM.

ILD is the most common complication in patients with IIMs, which has a prevalence of 78 % in IIMs patients. In addition, ILD is the major prognostic factor that contributes to mortality among PM and DM patients. Hence, the investigation with regarding to the relationship between FAM167A-BLK gene polymorphisms and ILD phenotype of PM/DM patients is of great significance. It is also worthwhile to note that the four SNPs (rs2736340, rs7812879, rs13277113 and rs2618479) were associated with PM/DM patients with ILD involvement. It is possible that the rs2736340, rs7812879, rs13277113 and rs2618479 variants may increase the susceptibility to PM/DM by affecting lung function. Therefore, whether PM/DM patients will develop to severe ILD, we might disclose the function of BLK gene, and then, we could predict the prognosis of PM/DM patients. The five SNPs (rs2736340, rs7812879, rs13277113, rs2618479 and rs2254546) resided in the same LD block, and they are the most common susceptibility loci in the FAM167A-BLK gene polymorphisms for other autoimmune diseases. Notably, the TGCAT haplotype was associated with PM, DM and combined PM/DM patients after Bonferroni correction, which confirmed that FAM167A-BLK gene play important role in the pathogenesis of PM/DM.

Because IIMs are rare disease, genetic association studies were always restricted, and early candidate gene studies only included small sample sizes and often divided clinical IIMs subgroups into PM, DM and myositis–CTD overlap syndrome in order to expand statistical power. Notwithstanding, our results were partly inconsistent with the outcomes of Caucasian and Japanese populations. Our study was the first comprehensive investigation involving patients of PM/DM from Chinese Han, and the clinical features of these patients were consistent with the international guidelines [25, 26]. Importantly, our sample size already has the power (more than 80 %) to examine moderate or even marginal associations. Yet there were some limitations to our present study that should be considered when interpreting our results. For example, we did not examine the function of these genetic variants in the development of PM/DM in these patients.

In conclusion, the present research was the first and comprehensive genetic association analysis of common variants in the FAM167A-BLK gene in Chinese Han population. And our results indicated that FAM167A-BLK gene polymorphisms (rs2736340, rs7812879, rs13277113, rs2618479 and rs2254546) were associated with the risk of DM in Han Chinese population.

## References

[1] Hirakata M (2007). Autoantibodies and their clinical significance in idiopathic inflammatory myopathies; polymyositis/dermatomyositis and related conditions. Nihon Rinsho Meneki Gakkai Kaishi.

[2] Chinoy H, Lamb JA, Ollier WE, Cooper RG (2011). Recent advances in the immunogenetics of idiopathic inflammatory myopathy. Arthritis Res Ther.

[3] Hassan AB, Nikitina-Zake L, Sanjeevi CB, Lundberg IE, Padyukov L (2004). Association of the proinflammatory haplotype (MICA5.1/TNF2/TNFa2/DRB1*03) with polymyositis and dermatomyositis. Arthritis Rheum.

[4] Pachman LM, Liotta-Davis MR, Hong DK, Kinsella TR, Mendez EP (2000). TNFalpha-308A allele in juvenile dermatomyositis: association with increased production of tumor necrosis factor alpha, disease duration, and pathologic calcifications. Arthritis Rheum.

[5] Mamyrova G, O’Hanlon TP, Sillers L, Malley K, James-Newton L (2008). Cytokine gene polymorphisms as risk and severity factors for juvenile dermatomyositis. Arthritis Rheum.

[6] Chinoy H, Salway F, John S, Fertig N, Tait BD (2007). Tumour necrosis factor-alpha single nucleotide polymorphisms are not independent of HLA class I in UK Caucasians with adult onset idiopathic inflammatory myopathies. Rheumatology.

[7] Chinoy H, Salway F, John S, Fertig N, Tait BD (2007). Interferon-gamma and interleukin-4 gene polymorphisms in Caucasian idiopathic inflammatory myopathy patients in UK. Ann Rheum Dis.

[8] Gono T, Kawaguchi Y, Sugiura T, Furuya T, Kawamoto M (2010). Interferon-induced helicase (IFIH1) polymorphism with systemic lupus erythematosus and dermatomyositis/polymyositis. Mod Rheumatol.

[9] Werth VP, Berlin JA, Callen JP, Mick R, Sullivan KE (2002). Mannose binding lectin (MBL) polymorphisms associated with low MBL production in patients with dermatomyositis. J Investig Dermatol.

[10] Chinoy H, Platt H, Lamb JA, Betteridge Z, Gunawardena H (2008). The protein tyrosine phosphatase N22 gene is associated with juvenile and adult idiopathic inflammatory myopathy independent of the HLA 8.1 haplotype in British Caucasian patients. Arthritis Rheum.

[11] Sugiura T, Kawaguchi Y, Goto K, Hayashi Y, Tsuburaya R (2012). Positive association between STAT4 polymorphisms and polymyositis/dermatomyositis in a Japanese population. Ann Rheum Dis.

[12] Miller FW, Cooper RG, Vencovsky J, Rider LG, Danko K (2013). Genome-wide association study of dermatomyositis reveals genetic overlap with other autoimmune disorders. Arthritis Rheum.

[13] Jani M, Massey J, Wedderburn LR, Vencovsky J, Danko K (2014). Genotyping of immune-related genetic variants identifies TYK2 as a novel associated locus for idiopathic inflammatory myopathies. Ann Rheum Dis.

[14] Sugiura T, Kawaguchi Y, Goto K, Hayashi Y, Gono T (2014). Association between a C8orf13-BLK polymorphism and polymyositis/dermatomyositis in the Japanese population: an additive effect with STAT4 on disease susceptibility. PLoS ONE.

[15] Hom G, Graham RR, Modrek B, Taylor KE, Ortmann W (2008). Association of systemic lupus erythematosus with C8orf13-BLK and ITGAM-ITGAX. N Engl J Med.

[16] Harley JB, Alarcon-Riquelme ME, Criswell LA, Jacob CO, Kimberly RP (2008). Genome-wide association scan in women with systemic lupus erythematosus identifies susceptibility variants in ITGAM, PXK, KIAA1542 and other loci. Nat Genet.

[17] Han JW, Zheng HF, Cui Y, Sun LD, Ye DQ (2009). Genome-wide association study in a Chinese Han population identifies nine new susceptibility loci for systemic lupus erythematosus. Nat Genet.

[18] Stahl EA, Raychaudhuri S, Remmers EF, Xie G, Eyre S (2010). Genome-wide association study meta-analysis identifies seven new rheumatoid arthritis risk loci. Nat Genet.

[19] Ito I, Kawasaki A, Ito S, Kondo Y, Sugihara M (2010). Replication of association between FAM167A(C8orf13)-BLK region and rheumatoid arthritis in a Japanese population. Ann Rheum Dis.

[20] Ito I, Kawaguchi Y, Kawasaki A, Hasegawa M, Ohashi J (2010). Association of the FAM167A-BLK region with systemic sclerosis. Arthritis Rheum.

[21] Lessard CJ, Li H, Adrianto I, Ice JA, Rasmussen A (2013). Variants at multiple loci implicated in both innate and adaptive immune responses are associated with Sjogren’s syndrome. Nat Genet.

[22] Nordmark G, Kristjansdottir G, Theander E, Appel S, Eriksson P (2011). Association of EBF1, FAM167A(C8orf13)-BLK and TNFSF4 gene variants with primary Sjogren’s syndrome. Genes Immun.

[23] Kikuchi Y, Koarada S, Tada Y, Ushiyama O, Morito F (2001). Difference in B cell activation between dermatomyositis and polymyositis: analysis of the expression of RP105 on peripheral blood B cells. Ann Rheum Dis.

[24] Krystufkova O, Vallerskog T, Helmers SB, Mann H, Putova I (2009). Increased serum levels of B cell activating factor (BAFF) in subsets of patients with idiopathic inflammatory myopathies. Ann Rheum Dis.

[25] Bohan A, Peter JB (1975). Polymyositis and dermatomyositis (first of two parts). N Engl J Med.

[26] Bohan A, Peter JB (1975). Polymyositis and dermatomyositis (second of two parts). N Engl J Med.

[27] Hochberg MC (1997). Updating the American College of Rheumatology revised criteria for the classification of systemic lupus erythematosus. Arthritis Rheum.

[28] Arnett FC, Edworthy SM, Bloch DA, McShane DJ, Fries JF (1988). The American Rheumatism Association 1987 revised criteria for the classification of rheumatoid arthritis. Arthritis Rheum.

[29] Preliminary criteria for the classification of systemic sclerosis (scleroderma) (1980). Subcommittee for scleroderma criteria of the American Rheumatism Association Diagnostic and Therapeutic Criteria Committee. Arthritis Rheum.

[30] Vitali C, Bombardieri S, Jonsson R, Moutsopoulos HM, Alexander EL (2002). Classification criteria for Sjogren’s syndrome: a revised version of the European criteria proposed by the American-European Consensus Group. Ann Rheum Dis.

[31] Sharp GC, Irvin WS, Tan EM, Gould RG, Holman HR (1972). Mixed connective tissue disease—an apparently distinct rheumatic disease syndrome associated with a specific antibody to an extractable nuclear antigen (ENA). Am J Med.

[32] Sontheimer RD (2002). Would a new name hasten the acceptance of amyopathic dermatomyositis (dermatomyositis sine myositis) as a distinctive subset within the idiopathic inflammatory dermatomyopathies spectrum of clinical illness?. J Am Acad Dermatol.

[33] Skol AD, Scott LJ, Abecasis GR, Boehnke M (2006). Joint analysis is more efficient than replication-based analysis for two-stage genome-wide association studies. Nat Genet.

[34] Purcell S, Cherny SS, Sham PC (2003). Genetic Power Calculator: design of linkage and association genetic mapping studies of complex traits. Bioinformatics.

[35] Barrett JC, Fry B, Maller J, Daly MJ (2005). Haploview: analysis and visualization of LD and haplotype maps. Bioinformatics.

[36] Dalakas MC (1991). Polymyositis, dermatomyositis and inclusion-body myositis. N Engl J Med.

[37] Dalakas MC, Sivakumar K (1996). The immunopathologic and inflammatory differences between dermatomyositis, polymyositis and sporadic inclusion body myositis. Curr Opin Neurol.

[38] Mastaglia FL, Phillips BA (2002). Idiopathic inflammatory myopathies: epidemiology, classification, and diagnostic criteria. Rheum Dis Clin N Am.

[39] Dymecki SM, Zwollo P, Zeller K, Kuhajda FP, Desiderio SV (1992). Structure and developmental regulation of the B-lymphoid tyrosine kinase gene blk. J Biol Chem.

[40] Tretter T, Ross AE, Dordai DI, Desiderio S (2003). Mimicry of pre-B cell receptor signaling by activation of the tyrosine kinase Blk. J Exp Med.

